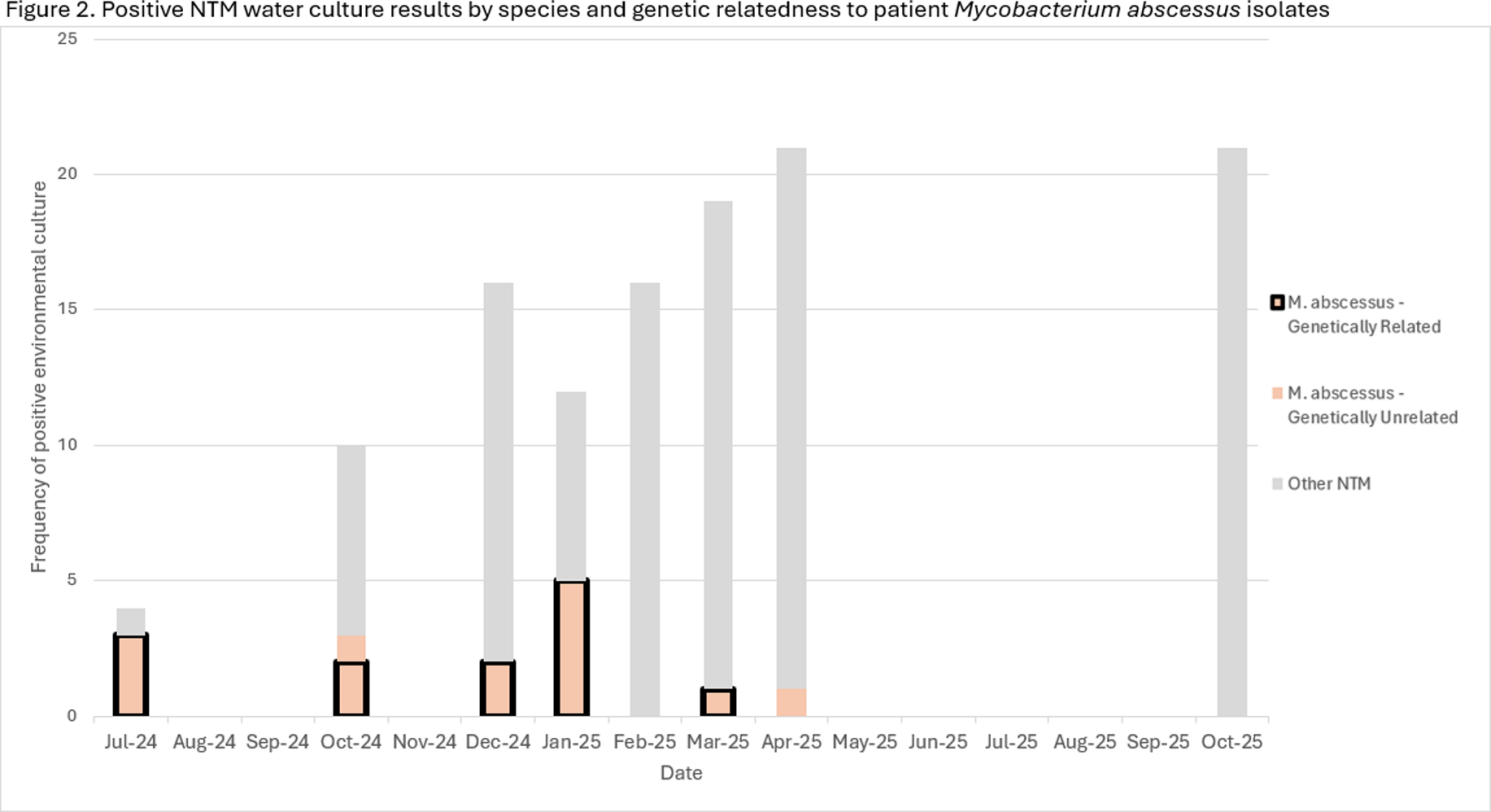# 41 Motivations for Clostridioides difficile testing algorithms among Veterans Affairs Medical Centers

**DOI:** 10.1017/ash.2026.10480

**Published:** 2026-06-23

**Authors:** Spencer Schrank, Alexander Sundermann, Ashley Ayres, Marissa Griffith, Kathleen Shutt, Kady Waggle, Lora Pless, Lee Harrison, Graham Snyder

**Affiliations:** 1 UPMC/University of Pittsburgh; 2 University of Pittsburgh; 3 UPMC

## Abstract

**Background:** Nontuberculous mycobacteria (NTM) are ubiquitously found throughout the environment, including healthcare facilities, and disproportionately affect immunocompromised hosts. We have a limited understanding of healthcare-associated transmission. Detailed outbreak investigations may yield insights into transmission risk. **Method:** An investigation of a Mycobacterium abscessus outbreak began after identifying a higher-than-expected frequency of infections among solid organ transplant recipients in September 2024. Cases were defined as individuals with a first-time positive M. abscessus culture from any anatomical site and a prior inpatient admission to our hospital. Our epidemiologic investigation included creating a case line list with potential exposures during the 90 days preceding microbiologic diagnosis, direct observations of patient care, a case-control study to identify potential point sources and transmission routes, and environmental cultures to test source/transmission hypotheses. Whole-genome sequencing (WGS) of case isolates was performed to confirm genetic relatedness (defined as ?10 single nucleotide variant difference). Prospective microbiologic surveillance was used to identify new cases. **Result:** Between May 2023 and the ongoing investigation through December 2025, 31 cases were identified. Twenty (65%) were solid organ transplant recipients, including 12 (60%) lung transplants (Figure 1, Table 1). Our epidemiologic investigation did not identify any other geographic, procedural, or point source exposures with commonality among <20% cases. The case-control study identified transplant status as a significant risk factor for transmission. We hypothesized transmission occurred through contaminated water exposure in inpatient rooms during routine care; environmental cultures demonstrated M. abscessus in potable water sources throughout the hospital (Figure 2). WGS identified 2 distinct clusters comprising 3 and 14 patients, with genetic relatedness to environmental M. abscessus isolates. Sequential flushing and chlorination of the facility-wide water system were performed without significant changes in the frequency of NTM isolation. To mitigate exposure risk, point-of-use filters were installed and tap water avoidance was enforced on units predominantly caring for patients with immunocompromising conditions. Following implementation of tap water avoidance, only 4 genetically related cases have been identified within 11 months of follow-up. **Conclusion:** Water exposure during routine patient care among susceptible hosts increases NTM risk. Eradication of NTM from aging plumbing systems is challenging; tap water avoidance combined with POU filtration appeared to have abated this outbreak. Infection prevention programs should consider routine monitoring of NTM infection rates, particularly M. abscessus, to detect a higher-than-expected frequency. Routine water quality surveillance should include NTM cultures. Studies are needed to identify effective methods of NTM eradication from potable water supplies.

**Figure:**
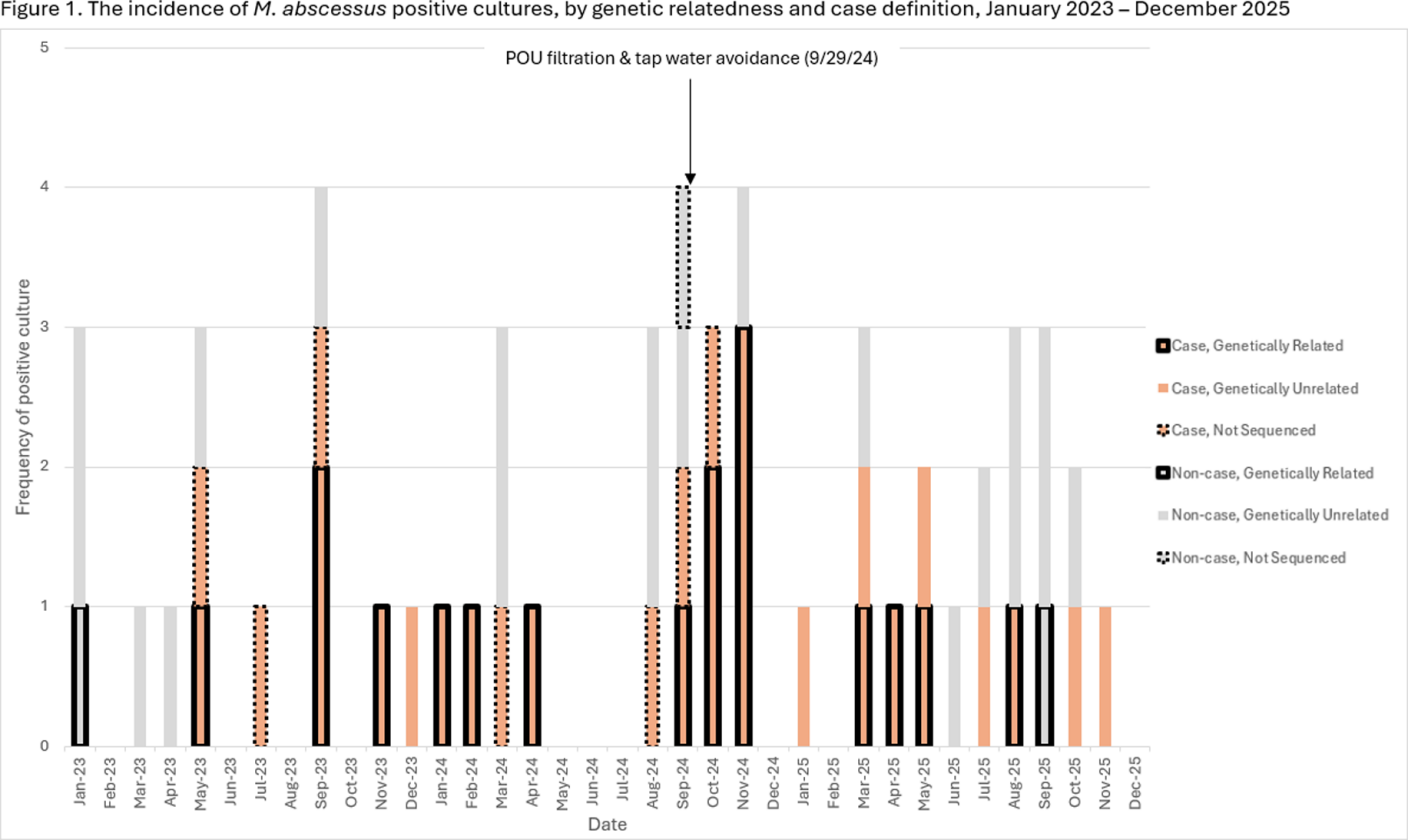

**Figure:**
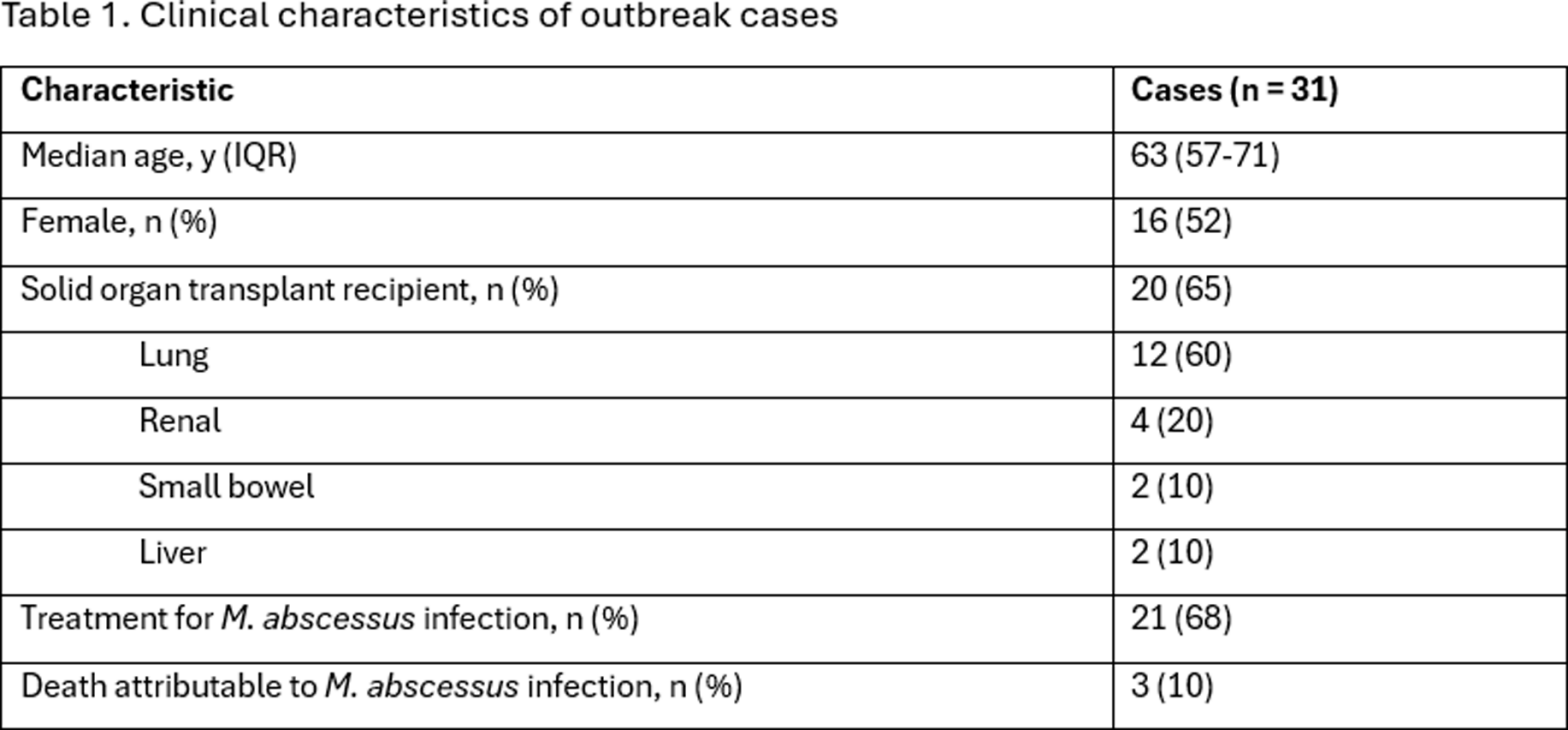

**Figure:**